# Fourteen-Year Patency of an Anterior Tibial Artery-Saphenous Vein Fistula in an Ambulatory Patient

**DOI:** 10.1155/2022/4135532

**Published:** 2022-12-05

**Authors:** Zerrin Pulathan, Gökalp Altun

**Affiliations:** Cardiovascular Surgery Department, Karadeniz Technical University Faculty of Medicine, Trabzon, Turkey

## Abstract

**Background:**

Ankle arteriovenous fistulas are the rarest vascular access type among lower extremity fistulas for hemodialysis patients with end-stage renal disease. Here, we present a case of a tibial-saphenous fistula that remained open for a long time despite a recurrent anastomotic aneurysm. *Case Presentation.* A 43-year-old female patient who had been undergoing hemodialysis via a right femoral tunnel catheter for six months was referred for recurrent catheter infection and a 4 cm pulsatile mass in the anterior aspect of the ankle. While she had been undergoing hemodialysis through a right tibial-saphenous fistula for fourteen years, hemodialysis continued after the fistula's closure due to total occlusion of the great saphenous vein through the tunneled catheter. After balloon angioplasty to the right subclavian vein, we performed right upper extremity basilic vein transposition. Later, after starting adequate dialysis from the basilic vein fistula and removing the femoral catheter, we performed a resection of the anastomotic aneurysm in the right ankle and repaired the anterior tibial artery. Because this is the only ambulatory patient and the one with the longest patency of ankle arteriovenous fistulas in the literature and the only case in which the anterior tibial artery was used, the case is presented and discussed in light of the literature.

**Conclusion:**

Despite many complications and low patency rates reported in the literature, ankle vessels should be considered for autogenous vascular access in selected patients.

## 1. Introduction

Upper extremity autogenous arteriovenous fistulas (AVF) are the best options for hemodialysis (HD). The most common indications for vascular access in the lower extremities are inadequate vascular structures in the upper extremities or bilateral central vein occlusion. Creating an autogenous arteriovenous fistula for the lower extremities is extremely rare, and usually, a saphenofemoral AV fistula is used [1]. Interventions below the knee are rare due to maturation problems related to inadequate dilatation of the great saphenous vein, frequent stenosis due to intimal hyperplasia, and ischemic complications due to diabetes and peripheral arterial disease [2, 3]. Typically, the posterior tibial artery is used in this body region. In our case, the fistula was formed between the anterior tibial artery and the saphenous vein.

## 2. Case Presentation

**Figure 1:**
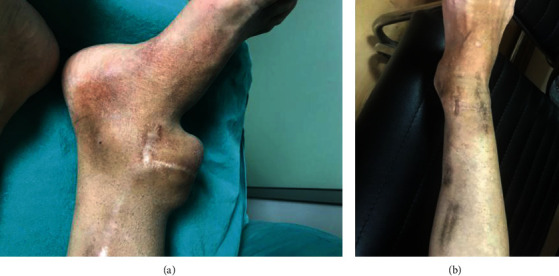
(a) Aneurysm and surgical incisions on right ankle (transverse scar is due to aneurysmoplasty operation performed nine years ago). (b) Image of the right ankle at the 3rd year postoperatively.

**Figure 2:**
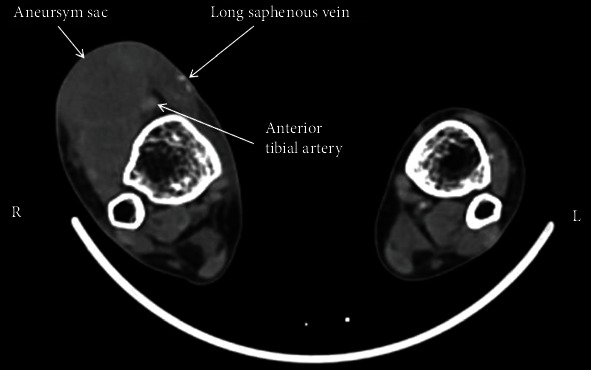
Computed tomography of the patient's right ankle aneurysm shows calcification in the saphenous vein and an intact aneurysm.

A nephrologist referred a 43-year-old female due to pulsatile swelling at the anterior part of the right ankle, pain, and recurrent infection of the femoral tunneled catheter. The patient was on hemodialysis for seventeen years, and hemodialysis had been performed through left radiocephalic, left brachiocephalic, right radiocephalic, right forearm prosthetic loop, and right brachiocephalic fistulas created in the upper extremities for three years. Then, the tibial-saphenous fistula was created fourteen years ago on the right ankle after the initial fistulas had become unusable. Hemodialysis was started on the below-knee saphenous vein two months after the creating that fistula. However, an ankle aneurysm developed three years later, and an aneurysmoplasty was performed in the fifth year after creating this fistula. After that, hemodialysis was effectively performed for another nine years (fourteen years in total). Although the swelling at the ankle worsened, the fistula was used without further intervention, as long as it was functional. When the great saphenous vein was occluded while the patient was being followed in another center six months ago, a tunneled catheter was placed in the right femoral vein, and hemodialysis was continued without any invasive or surgical procedure to render the fistula functional. Physical examination revealed a pulsatile mass on the anterior aspect of the right ankle, transverse and longitudinal surgical incision scars on the aneurysm sac and medial malleolus, pigmentation secondary to cannulation of the great saphenous vein, an increase in calf circumference of 1 cm, fibrotic thickening, and thrombosed aneurysmal dilatation on the great saphenous vein (Figure 1). There was no palpable thrill over the pulsatile aneurysm sac. The posterior tibial pulse was strong; the anterior tibial pulse was weak, and there were no findings suggestive of ischemia. Lower extremity arterial Doppler's ultrasonography revealed a 41 × 45 mm nonthrombosed aneurysm sac at ankle level in the right anterior tibial artery and a biphasic flow pattern in the distal artery. In venous Doppler's ultrasound, the deep venous system was patent; the saphenous vein was occluded entirely at the level below the knee, and the diameter of the proximal saphenous vein at the level above the knee was 3.4 mm. An aneurysmatic segment with a diameter of 45 mm was detected in computed tomography angiography (CTA) examination of the right ankle (Figure 2). Physical examination and upper extremity Doppler's ultrasonography revealed that only the right basilic vein was open with an appropriate diameter (3.5 mm) to create a new AVF. Upper extremity venography performed due to dilated collateral veins around the shoulder showed stenosis of the short segment of the brachiocephalic vein, and complete patency was obtained by balloon angioplasty. A functional AVF was obtained after right basilic vein transposition (BVT), and HD was initiated 34 days later. The tunneled catheter in the right femoral vein was removed, and surgical repair of the ankle aneurysm was performed one week later. A 4 cm incision was made on the anterior tibial artery trace in the ankle. After vascular control was achieved, the aneurysm sac was excised, and the artery was repaired with 7/0 Prolene sutures laterally. Anterior tibial pulse was palpable after the surgery. Hemodialysis has continued for three years via the right BVT, thanks to balloon angioplasty repeated twice in the right brachiocephalic vein. The patient is still on the transplant list and waiting for a donor.

## 3. Discussion

Creation of a lower extremity saphenous arteriovenous fistula is quite rare and is usually performed above-knee level. The ankle region is rarely chosen due to the tendency towards trauma, arterial calcifications frequently seen in patients with kidney disease, and possible intolerance of the distal vascular bed due to diabetic angiopathy or peripheral artery disease. In addition, the wall structure of the saphenous vein and compression of the surrounding fascia may cause insufficient dilatation and maturation as well as increased neointimal hyperplasia. Kahraman et al. reported that they could initiate hemodialysis in only three (27.3%) of eleven tibial saphenous fistula cases in which they applied balloon dilation to the saphenous vein and its fascia while creating the ankle fistula [2]. Also, lower extremity edema, which may be caused due to volume burden or impaired venous circulation due to fistula, may lead to difficult cannulation. In the long-term, complications such as infection, steal syndrome, thrombosis, and aneurysm caused by the fistula cause a greater risk of extremity loss in this region than in other areas. No complications other than an aneurysm developed in the long-term were observed in this patient, which may be due to the patient's age and the absence of other comorbidities such as obesity, diabetes, hypertension, and peripheral arterial disease [1, 3]. If indicated, fistula functionality can be prolonged with interventions such as aneurysmorrhaphy, aneurysmoplasty, and remodeling in aneurysmatic fistulas. Although primary and secondary patency rates, infection rate, and incidence of critical ischemia for autogenous fistulas of the femoral region for one year were 83%, 93%, 1.6%, and 21%, respectively, literature data for patency and complication rates of ankle fistulas is quite limited [1]. Flora et al. reported that thrombosis developed in one of the three cases with tibial-saphenous AV fistula in the fourth month [4]. In the other two patients, AV fistulas remained functional after a mean follow up of twelve months. Goh et al. reported that they formed a posterior tibia-saphenous AVF in a 50-year-old female patient with central vein occlusion, and cannulation was performed with a flow of 300 ml/min at the twelfth week. It was stated that anastomotic thrombosis developed in this patient five months later; the AVF was moved more proximal and remained functional for six months [5]. The longest patency in the literature was reported as 24 years in a 63-year-old paraplegic patient. Rim et al. reported no complications related to the fistula in this paraplegic patient, but they did not recommend this method because of the risk of trauma in outpatients [6]. Hara et al. reported the second-longest patency [7]. In this case, the posterior tibial artery-saphenous vein fistula remained open for fifteen months until kidney transplantation. Our case is a female patient who could perform daily routine activities for fourteen years until the fistula occluded. She is the first mobile patient with the longest duration of patency. Most of the cases in the literature are posterior tibial artery-saphenous vein fistulas. Our case is the only anterior tibial artery-saphenous vein fistula case in the literature to the best of our knowledge. In our case, 14-year fistula patency can be attributed to the aneurysmorraphy intervention performed nine years ago and presented by Günday [8]. Inston et al. reported that the one-year patency rate of fistulas whose aneurysms were treated was over 80% [9]. A basilic vein transposition is a good option for vascular access due to high maturation and patency rates in patients with insufficient superficial veins [10]. The rarity of imaging and intervention methods in previous years may explain why BVT fistula had not been tried in this patient earlier. Imaging central veins and performing angioplasty in patients with shoulder venous collaterals may allow upper extremity AVF patency and create more complex fistulas like BVT.

## 4. Conclusion

Despite the limited case series in the literature and uncertain long-term patency rates, ankle AVFs should be considered as an alternative autogenous vascular access route in selected patients.
